# Impact of green finance and fintech on sustainable economic growth: Empirical evidence from India

**DOI:** 10.1016/j.heliyon.2023.e16301

**Published:** 2023-05-15

**Authors:** Sreenu Nenavath, Shashwat Mishra

**Affiliations:** Department of Management Studies, Maulana Azad National Institute of Technology Bhopal, India

**Keywords:** Green finance, Fintech, Economic growth, Quality environment and finance structure

## Abstract

This research study analytically investigates the influence of green finance and financial technology on sustainable economic growth. The analysis is based on data from Indian states from 2010 to 2021. The research paper uses the panel regression method to examine the association between fintech, green finance and economic growth by applying a two-step GMM (generalized model of moments) to determine the endogeneity issues of the variables. This paper reveals that green finance widely helps quality economic growth by significantly impacting finance structure, financial effectiveness, and environmental quality protection development. Furthermore, fintech enhances the significant effect of green finance in the finance structure and environmental quality protection while lacking consequences on the association between green finance and economic effectiveness. Based on the results, the current research paper offers policy submissions for policymakers and the Government of India, including strengthening the consolidation of fintech growth with green finance, structuring a quality environmental revelation outline to control state governments in refining the effectiveness of green finance, and emerging prolonged satisfactory protocol as an outside involvement proceeding to encourage green finance in the non-public sector.

## 1 Introduction

In recent decades, the economic growth of India has increased enormously due to its new economic policies made by the government for the proper utilization of available environmental resources. Therefore, the over-exploitation of forest resources caused environmental deterioration and contamination that consider a serious challenge to protect the environmentonan urgent basis and also to prevent several negative impacts on human health [1]. Hence, currently in India, the channelling of the resources towards green ecosystem had planned to provide the needs of the local people living in rural areas, because their livelihood mostly depends on local available environmental resources. Because of energy, currently, fossil fuels have gained more attention becausethey fulfil almost around 60 various energy demands in India. Therefore, high consumption of fossil fuels also releaseda substantial amount of toxins into the environment that has damaged the ecosystem.

Many researchers have been working on the decline in thedegradation rate of the environment by different methods [2]. In addition to that the Indian government has been working on making sustainable goals for the country by adopting measures that promotehigh-calibre economic growth and eco-friendly techniques. The green finance strategy to fund various projects that ensure they fulfil the above constraints plays a pivotal role inthe sustainable development of the worldwide economy.

Green finance mainly concerns two broad areas finance and the environment; its product has gained recognition and helped to deliver high-quality development for various fields in different parts of the world. In owing to that India issued $6.11 billion worth of green bonds in 2021and set another $3.3 billion in 2022 as it transitions towards a low-carbon economy. Green Finance have several advantages such as it seeks to offer operating, investing, financing of funds, and supplementaryservicesto the finance industry, which also provides the project with an outlook. However, Carbon finance starts late but progressesfast in India, which is essential in launching a tough carbon trading market. Although green finance has become mostprominent in emerging economies, it is still unclear how much it contributes to high-quality economic development [3]. In addition, thatfintech in India has also performed exceptionally well in the last few years since its emergence. The adoption of financial technology among consumers and businesses has been expanding rapidly. The development of financial technology is anticipated to be vital in the nation's progression to a sustainable financial system and high-quality economic growth. It provides avenues for retail investors to explore and invest in products that suit the objective of environmental sustainability coupled with high-quality economic development. However, there exists a gap in the supply and demand for funds in renewables, there is an urgent need toaddress the problem of overcomingthe challenge [4]. It accelerates the growth of green finance by reducing information unevenness for investors and stakeholders, improvingefficiency, appreciating nature assets, and promotingsustainable lifestyles to support quality economic growth [5]. Green finance is associated with high-quality and sustainable development and these are important factors of economic growth; it also has sustainable expansion characteristic that offers a basis for planning closely associated with high-quality sustainable development. In addition to that, it also addsan essential factor of economic growththat offers a foundation for planned prosperity [6]. Green finance is concerned with concerning public and private financial structures with eco-friendly business models which are directed at clean energy, enhanced water and air quality, disposable plastic and so on; and green finance fills gaps among investors, lenders, consumers, business owners and all stakeholders.

The objectives of the current study add to the corpus of knowledge in the following areas and make several contributions to the literature on green finance. First, it contributes by shedding light on green finance and honouring academic research to determine how green finance affects high-quality economic growth. While past studies on green finance research emphasized a particular environmental aspect of economic development, Itspecifically explores the impact of green financing on comprehensive, high-quality economic development. Second, this studyconsiders a recent call for attention to the contribution of fintech to the growth of green finance. Identified that there is not enough study on fintech that how it altersthe connection between green financing and economic development. Therefore, considered fintech a moderator variable and planned to investigate its covert influence on the association between green finance and high-quality economic growth. And alsodevelopment entry provides a genuine understanding of the development impact of green finance. The above-stated fintech results could offer significant fiction for the stakeholders so they can redesign economic development by making progress in green finance and financial technology.

## 2 Literature review

Green finance explains that the “financial funds flowing into sustainable progressventures and initiatives, environmental products, and policies that encourage the development of a more sustainable economy” [7]. Notably, while making investment and financing decisions, financial institutions take environmental protection into account, which drives cash to green industries. This approach contemplates the adverse environmental impacts and how we should prepare ourselves to strike a balance between excessive natural resource exploitation and economic growth. Eargrowth, the financial objectives only considered a unidirectional approach to maximizing the returns ignoring the environmental impacts [8]. Green finance bridges the gap wherein variables are supposed to be to ensure sustainable economic development. Green finance helps in channelizing resources more efficientlyand efficient environmental protection [11]. Studies suggest that green finance contributes to high-quality economic development by positively impacting the ecological environment, economic efficiency, and economic structures [9]. Fintech is a combination of financial services delivered using technology. It brings abundant opportunities as the technology helps organizations in the financial services sector to reduce friction and hence solutions much more effectively. However, the impact of this technology on building a high-quality, sustainable framework for the development of the economy is a topic of research as it is in a nascent stage, and we are yet to capture its impacts. High-quality economic development is achieved with the help of many factors. According to studies, countries have exploited natural resources at an unprecedented rate to achieve sustained economic growth because most of the traditional industries lacked both technological innovation and energy efficiency, resulting in severe pollution [[8], [10]]. However, if economic growth is achieved through renewable energy production, it has a lasting positive impact on the environment and economy [9]. It is noticed that high-quality economic development growth is multifaceted as it has impact factors like technological innovation, organisational factors, financial factors, and political factors.

### 2.1 Fintech and its impact on green finance

Fintech refers to state-of-the-art solutions to upgrade and mechanize the delivery and use of financial services. It is significant in finance as it completely changes how things are in the Banking, Financial, Services, and Insurance sectors. Fintech in India is the 3rd largest in the world after China and USA. It is expected to overgrow at a CAGR of 24.57% per annum. Fintech acts as a facilitator for innovations in the financeindustry, catering to both products and services. Evidence from countries like China suggests that growth in fintech innovation contributes to green credit, investment, and areas that stimulate green growth. Also, the green development of the economy goes hand in hand with innovations in science and technology, which cannot be done without improving efficiency [9]. Fintech primarily involves technologies applied in the financial services sector, including mobile payments, money transfers, loans, fundraising, and asset management. It has been growing expeditiously, and the infrastructure to support it is also developing. Fintech establishment is more straightforward in advanced economies with established market rules, private equity, and fundamental infrastructure [12]. Because, in the case of well-developed economies, already have the basic enabling infrastructureto support the advancements. Studies also show that internet servers, mobile phone subscribers, and workforce availabilitybenefit the development of fintech. Financial technology makes it easier for investors, especially private ones, to access new sources of funding and investment [13]. It also comes to our knowledge that sufficient servers, mobile subscribers, and workforce contribute positively toward the growth of financial technology. Studies found that the difficulty in obtaining essential financial services like loans leads to the emergence of new fintech businesses in a nation [14]. The system may theoretically become more effective with a more extensive user and more significantand new fintech initiatives [10].

However, we cannot ignore that technical innovation is one of the critical factors responsible for environmental pollution and economic growth. Also, it provides developing countries with ample chances for economic growth. But technical innovation is a doubtful advantage for the economy and environment. On the one hand, it can provide opportunities for economic growth with increased production efficiency of the enterprises and reduced production costs. It can also help us break the financial users for the sustainable development of the economy as it leads to production efficiency. On the other hand, it increases the demand for natural resources damaging sustainable economic growth [15]. The impact of fintech in constituting a green economy is peculiar. Technology advancement and innovation effectiveness of organizations in acquiring knowledge regarding credit which reduces financial friction with the help of information symmetry. Since, fintech innovation focuses on digitally transforming technologies like Big Data, IoT,AI, cloud computing, and blockchain. Studies show that fintech innovation accelerates the evolution of the ecosystem by expanding financial aid, enhancing resource allocation, streamlining resource allocation, and helping in reducingeducationfinance [16].

Financial technology makes it easier for people to obtain new financial resources from a bigger group of investors. It also pointed out that green bonds are effective investments; it reduces a company's carbon footprint and creates long-term value. An application of fintech could be agriculture. It can transform the processes into sustainable funding and distribution methods for sponsoring a project or business enterprise by collecting money from several people, usually online and through electronic payment systems [14]. Financial technology can help the stakeholders in mobilizing funds into environmentally friendly and environmentally close avenues and applications that can be expanded to make people more sensitive towards their carbon footprint and what they can do to reduce it. A similar application like ANT Group in China (the fintech company in China) has been developed. It tracks human activities and rewards them when they make choices that benefit the environment, which plants trees on their behalf. This way it brings efficiency to the financial world and the environment, which brings real change to society.

### 2.2 Green finance and economic development

Green finance provides a set ofenvironmentally beneficial instruments ensuring sustainable economic development. Research in this area is at an early stage; earlier studies have highlighted the short-term view of the organizations to achieve growth. The green finance mechanism bridges the gap of the traditional finance system, which only s the projects' profitability by developing a broader purview, including protecting the environment and energy efficiency [9]. Studies have analysedsignificantobstacles to green investment for sustainable energy infrastructure and policy intervention, which shows that uncertainty of the policies, along with short-termism in the financial system, are the two significant investment barriers [17]. It is also noticed that the beneficial effects of green finance on enterprises, as it accelerates innovation in firms which helps the economy switch to green and efficient production [18]. Researchers have also started to pay attention to the development of sustainable finance to ensure high-quality economic growth. Sustainable development helps form a society of happy people by sustainably developing the economy and taking care of the organisation's financial goals [16].

Studies show that investments in the energy conservation and environmental protection sector can alter the industry's system by employing sustainable financial methods [18]. “The study investigated the relationship between urbanisation, hydel power use, and real gross domestic product in China and India from 1965 to 2013” [34]. It was argued that while hydel energy consumption has a short-term negative impact on emissions in both countries, urbanisation has a long-term favourable influence. According to existing knowledge, prior studies analysed the effect of green financing on economic development primarily from a unique environmental component or one incorporated index of many factors. Evidence from India suggests Green Finance policies are crucial in channelling funds to the less developed financial ecosystem. India has a lot of potential to leverage existing technology and relationships between banks and enterprises. With this comes an opportunity to direct the attention toward green finance. Green finance and firm innovation have a positive correlation [15], which means it plays a crucial role in the transition to improved and sustainable financing methods for manufacturing. Environmental Kuznets Curve shows that, to an extent,a linear relationship exists between CO2 emission and economic development. However, beyond a point, it starts to come down. Specifically, EKC shows a U-shaped relationship between economic growth and CO2 emissions. In the long run, it does not show any conflicts as the emissions decline with a sustained increase in high-quality economic development [19].

Studies suggest variations across cities regarding the number of resources used, the benefits to society and the economy, and environmental metrics influencing green growth [20]. R&D expenditure may not look conducive in the short term but it brings benefits in the long run. Considering the binding nature of R&D it is of utmost importance for the growth of a high-quality economy. It is also noted that Foreign direct investment positively affects the economy [21]. Studies suggest that Economic development should be studied in a detailed manner considering various impact factors of high-quality economic growth, with each indicator hand-picked and assessedindividually, which would provide us with a much more thorough understanding [22]. Some financial technologycompanies are dynamically integrating a green financial system with the objectives of adopting technology to decrease CO2 emissions and encourage effective resource use. For example, Paytm, PhonePe, MobiKwik, and PayU, are the largest fintech companies in India, with majorities of fintech companies in the mobile app that boosts customers to contribute to sustainable finance ventures. Moreover, it encourages users to engage in CO2-reducing behaviours like walking and using public transportation. As part of the desert recovery initiative, end users can grow a virtual tree by collecting carbon savings and earning green energy. It will eventually grow into a real tree. However, there is a research lacuna concerning the participation of fintech firms in environmental quality protection efforts in India. Explicitly, financial technology companies might not dynamically contribute to these endeavours. Hence, the outcome of financial technology in fast-tracking the shift of green finance to sustainable economic development in India is indistinguishable. For this paper, the role of fintech is examined as a borderline situation that can control the outcome ofsustainable finance on sustainable economic growth [33].

## 3 Research hypotheses

This research paper aims to elucidatehow green finance determines sustainable economic growth. The multiple hypotheses concerning the associations between green finance and sustainable economic development are explained. The paper assesses sustainable economic growth from three characteristics created as three parameters, i.e., economic system (ES), economic effectiveness (EE), and Environmental Quality Management (EQM). It suggests green finance's critical positive effect on sustainable economic growth characteristics. The study also examines the regulating impact of financial technology on the associations between green finance and sustainable economic growth. The comprehensive hypotheses are explained as follows.

### 3.1 Impact of green finance on environmental quality, economic efficiency, and system

The study examines that green finance contributes to the development of environmental quality as it affords provisions and direction for stakeholders that can accomplish a mutually beneficial condition between economic feasibility and environmental quality protection [35]. Initial, green finance support companies with a green organization approach to get access to substantial loan size [21], which boosts companies’ green conversion from high energy consumption and high smoke, dust, or pollution to a much more sustainable approach. Subsequently, the green investment structure empowers stakeholders, such as enterprises, to control the entire procedural course of the investment scheme and assurance that the investment venture encounters green values [22]. Lastly, green finance supports the utilization of renewable fuel (hydroelectric, geothermal, solar, wind, and biomasses energy) fuel use in customer groups [23]. In the meantime, financial organizations could regulate the credit in acquiring green products (for example, electric vehicles). In this connection, with the help of the green finance approach and environmental quality protection, the study developed the hypothesize that:

H1a Green finance is positively correlated to environmental quality protection.

The present research paper investigates whether green finance helps improve economic efficiency in production, allocation, and distribution. (1) Equated with the conventional finance system, green finance highlights the operative use of capital [20], which establishes significant standards for measuring the efficiency of its actions. In this connection, with an additional green finance structure, extra labour productivity, land productivity, and other capital will be put into this arena, which can be highly efficiently used for resourceful economic growth. (2) The green finance instruments choose the green project and control the investment flow to sustainable product manufacturing with lesser funding expenditure and, more significant asset payback [24]. Indian banks play a significant role progressively foremost in the distribution of green finance in the credit market [25], enabling green finance venture construction and raising the competence of the project submission. Consequently, with additional capital paying attention to green manufacturing growth, it will accomplish a righteous sequence of more effectiveness of economic growth. Henceforth, the hypothesis is framed based on how green finance improves economic efficiency:

H1b Green finance is positively associated with high efficiency of economic development

The green finance system has a positive effect on enhancing the economic system. The infrastructure projects maintained by green finance have a comparative policy situation [17]. With these rewards, it benefits to interest enormous volumes of capital to green finance projects, to enhance the venture construction to additional biodegradable productions. Moreover, green finance is demonstrated to leverage the evolution of more pollution and energy-consuming organizations into green manufacturing finished using to limit credit policies [26]. The financial system can be improved and promotedwith more companies transitioning to green manufacturing. Consequently, the hypotheses developed:

H1c Green finance is positively correlated to the development of the economic system

### 3.2 Impact of fintech on environmental quality, economic efficiency, and SSystem

Fintech (financial technology) and modern technologies augment pioneering financial products and facilities [27]. Based on ABCD, AI (“Artificial Intelligence”), BD (“Big Data”), CC (“Cloud Computing”), and BT (“Blockchain Technologies”), financial technology illustrations are immensely capable of enlightening financial facilities in effectiveness [11]. In this paper, financial technology delivers a limited form of services that impact the efficiency of green finance on sustainable economic growth [29]. Due to this reason, financial technology can encourage green finance by organizations, investors, and other stakeholders with an approachable, resourceful, crystal clear, and computerized green finance approach and facilities [26]. Explicitly, sustainable economic progress strains a high quantity of available finance into supportable infrastructure development, which does not match the mandatory scale [27], indicating the green finance research gap. To report this research lacuna, green finance can combine with financial technology to expand the approachability of sustainable projects. Predominantly, fintech has significant returns of a better regulator of customers’ finance, quick resolution, and operation, permitting leading business communications in any probable circumstance [30,31]. In this logic, financial technology may benefit environmentally quality protection-related firms acquiringbenefit environmentally quality protection-related firms acquiring investment capital quicker, inexpensively, and more reasonably. Furthermore, the significance of elasticity and effectiveness of financial technology enables the growth of green finance and reinforces its constructive effect on sustainable economic growth [32]. Hence, the study has developed the hypothesis that:

H2a Fintech significantly balanced the impact of green finance on environmental quality protection

H2b Fintech significantly balanced the impact of green finance on economic effectiveness

H2c Fintech significantly balanced the impact of green finance on the economic system

## 4 Research methodology

### 4.1 Model developed

The current paper examines the effect of green finance and financial technology on sustainable economic growth; the regression equation of the model is as follows:

(1) Sijt = μ0+ μ1G_Fit+ μ2F_Tit+ μ3 G_Fit* F_Tit+ μ4CVit+εit

From equation (1), where Sijt denotes the price of sustainable economic growth pointer, j indicates the development of Indian states, I in the t the year. The G_Fitsuggests the level of green finance development in India during the study sample period, G_Fit indicates the fintech (Financial Technology) strength of Indian states during the study sample period, and CVit shows the price of control variables. G_Fit* F_Titshows the interface terms, where independent and control variables were homogeneous, before calculating the multiplicative standings to reduce multicollinearity. μ0 is the persistent time, μ1, μ2, μ3, and μ4 are coefficients of the regressor, moderator and control variables, correspondingly. ε is anarbitrary error term.

### 4.2 Selection of dependent, independent, moderating and control variables

#### 4.2.1 Dependent variables

In this study, sustainable economic growth comprises three indicators -financial system (ES), economic effectiveness (EE), and Environmental Quality Management (EQM). The paper created these three indicators based on the work of (Xu T (2018) and followed the classification and data accessibility principles. Environmental quality Management (EQM) denotes that the way of realizing economic evolution is biodegradable and source protection. Economic effectiveness (EE) discusses the rational formation of capital and enhancement of factor productivity. Eventually, an economic system (ES) states the apportionment of funds and the amount of personnel in three financial aspects, together with industry, investment, and economic effectiveness. The comprehensive measurements are listed in Table 1.

**Table 1:** Measurements of sustainable economic growth.

Stage −1	Stage –II	Stage –III	Explanation
sustainable economic growth	an economic system (ES)	Financial system (FS)	Total foreign investment/GDP
		Investment	ROI = Net income/Cost of investment x 100
		Trade openness	The ratio of exports plus imports over GDP
		Industrial growth	Market size/no of year
sustainable economic growth	economic effectiveness (EE)	Land Productivity	Crop output/land area
		Labour Productivity	Total Output/Total Input
		Capital Productivity	GDP/total investment
		Total factor productivity	Total factor productivity *capital input *labour input
sustainable economic growth	Environmental quality Management (EQM)	discharge of Industrial waste per unit GDP	releaseof Industrial wastage/GDP
		Sulphur dioxide (SO2) emissions per unit GDP	SO2/GDP
		Carbon Dioxide (CO2) Emissions per Unit of GDP	CO2/GDP
		Forest area (% of land area)	–
		The coverage ratio of the green space index	–

#### 4.2.2 Regressor

The paper carefully chooses green finance parameters to develop the green finance index. The reference to Ref. [28], green finance is classified into five subparts:

(1) carbon finance, (2) green insurance, (3) green credit, (4) green securities (5) green investment. The five classified features of green finance are created as follows:

Carbon finance is quantification by the proportion of CO2 emissions to gross domestic product. The primary source for the CO2 emission is coal, oil, and natural gas, and computed with the help of the following formula: CO2 emission = μ1β1coal+ μ2β2 natural gas+ μ3β3 petroleum. The International PanelInternationalPanelInternational Panel develops the CO2 emission constant μ and normal coal adaptation constant β produces the CO2 emission constant μ and normal coal adaptation constant β creates the CO2 emission constant μ and normal coal adaptation constant β on Environment Change (IPEC). Green insurance protection can be measured by ecological smog responsibility insurance. However, insurance coverage in India is still in its initial stages, and the evidence exposed is inadequate. The green credit facilities mainly discuss the green payment and loans of SMEs [22]. They are computed by utilising the sum of green credit of registered firms classified by the sum of credit of listed firms. To consider the green securities, the paper applied the share between the TSMV (“Total Stock Market Value”) of listed firms in the environmental quality management industry listed in Indian states and the TSMV of listed companies to calculate the green securities with the help of the study sample period. Green investment projects denote the way of source apportionment to ecological pollution resistor, targeting to decrease the environmental impairment of a firm's operation and comprehend sustainable growth. In this research paper, we emphasize the public sector; consequently, the green venture is set as the ratio of India's fiscal disbursement on energy management and environmental quality safety to India's total budgetary outflow.

#### 4.2.3 Moderator variable

Financial Technology can be determined as the strength of the innovative financial technology projects of Indian states during the study sample period. Financial technology firm strength explains the side by side of fintech activity and growth in the rural area. The paper sums up the number of anew initiated financial technology projects as the worth of financial technology strength.

#### 4.2.4 Control variables

In the meantime, economic growth can be affected by the variance in trade and industrial building among states; the paper applies energy consumption data of the previous year to control the outcome of contention in an industrial building on economic growth. GDP per capita apprehensions the inclusive state's economic growth level, connecting to (1) environmental governance, (2) innovation technology investment, and (3) resource allocation. The paper practices the log of selected variables in a direction to an explanation for its skewed distribution. Education statutes are pertinent in inducing the effectiveness of economic growth [29] and utilize the proportion of education outflow to gross domestic product to the portion of education level.

The researcher used two techniques to create the indicators of green finance and sustainable economic growth in this study. For green finance, this study uses the Generalized Principal Component Analysis to compute the green finance growth index for different states and metropolitan cities in India from 2010 to 2021. The validation of implementing this technique can investigate time series data and the PCA (principal component analysis). To that end, the paper used the Kaiser-Meyer-Olkin test to regulate in-case the data can be investigated by applying the Generalized Principal Component Analysis method. The outcome of the KMO assessment is 0.648 (>0.6), which specifies a robust correlation among the variables. Hence, the data can be investigated by applying the Generalized Principal Component Analysis method. The data on environmental quality protection, green finance, green coverage, and carbon finance were collected from the bacterial exopolysaccharides properties and structures database (“http://www.epsdatabase.com/database.html). Economic effectiveness and economic system-related data were collected from the Indian Statistical Department (2022). Green investment, green credit, and green securities were attained fromthe Wind Economic Database (“https://www.wind.com.cn/en/edb.html”). The data related to fintech (financial technology) were collected from the Indian fintech database (https://www.fintechdb.com, https://fintech.rbi.org.in). The descriptive statistics of variables are presented in Table 2.

**Table 2:** Descriptive statistics.

Variables	Mean	S. D	Minimum	Max	Samples
EQM	0.45	0.13	0.09	0.68	328
E-E	0.16	0.06	0.05	0.56	328
E-S	0.13	0.05	0.02	0.53	328
G-F	2.37e-7	0.62	−0.84	1.01	328
F-T	123.34	327.27	0	3172	328
E-C	5.11	0.27	3.63	5.57	328
GDPPC	7.31	0.32	5.16	7.02	328
Edu %	0.02	0.01	0.01	0.07	328
F-E	0.16	0.07	0.07	0.47	328

### 4.3 Data analysis and interpretation

A variance inflation factor (VIF) is a portion of the total of multicollinearity in regression examination. Multicollinearity exists when there is a correlation between multiple independent variables in a multiple regression model. This can adversely affect the regression results. In Table 3, We determined that for multicollinearity to confirm, it is not the main apprehension. The maximum VIF (“Variance Inflated Factor”) is less than the selected variables' threshold from Table 3. The outcome of the Variance Inflation Factor indicates the nonexistence of the multicollinearity factor in this paper. Before investigating the regressed analysis of the panel data, URT (“Unit Root Test”) and CT (“Cointegration Test”) are essential to confirm the reliability of the regression outcomes. The current paper applies the Hausman test and the Im-Pesaran-Shin (“IPS”) test to validate the stationarity of the data with the sample duration period and Indian state growth rate. In count, the paper uses the Kao test and Padroni test to confirm whether there is a long cointegration association is there or not.

**Table 3:** Detecting multicollinearity using VIF (variance inflation factor).

Variable	Variance Inflation Factor	1/Variance Inflation Factor
Green Finance	1.23	0.35
Fintech	0.89	0.57
Energy consummation	0.97	0.41
GDP per capita	1.17	0.32
Education %	2.13	0.21
Fiscal expenditure	2.37	0.18
The mean value of VIF	1.63	–

This research paper uses Stata SE 17 to execute the Harris–Tzavalis (“HT”) and Im–Pesaran–Shintests to determine the stationarity of the series on each variable to verify the validity. From Table 4 output, the unit root test highlights the financial technology, and gross domestic product per capita shows the non-stationary of the data. The article applies the unit root test to the first-order difference value. In the outcomes illustration, the variables show results that there is no unit root order at a 1% significance level. The development signifies that all non-stationary categorizations are first-order on their whole number stationary after action for first-order differences. More tests are required to observe if there is a long-durationsymmetry association and cointegration among the variables.

**Table 4:** Output of stationarity test.

Variable	Methods				Stationary test
	IPS Test	*P*-value	Hausman test	*P*-value	
economic system	−0.97	0.067	0.204	0.02	stationary
economic effectiveness	0.83	0.071	0.312	0.00	stationary
Environmental Quality	−1.17	0.001	0.251	0.00	stationary
Green finance	−2.741	0.610	0.319	0.01	stationary
Fintech	0.728	0.002	0.729	0.05	Non-stationary
Energy consummation	−1.84	0.131	0.631	0.03	stationary
GDP per capita	−0.82	0.024	0.614	0.27	Non-stationary
Education %	−0.36	0.011	0.371	0.00	stationary
Fiscal expenditure	0.80	0.36	0.520	0.06	stationary

From Table 5, the researcher uses the Kao panel cointegration, Peroni panel cointegration test, and Peroni panel cointegration tests on the variables (economic system, economic effectiveness, and environmental quality protection). The Kao test and Pedroni panel cointegration test outcomes are presented in Table 5. Meanwhile, the panel cointegration test outcomes all permit the 5% significance level; it can be conditional that there is a panel cointegration association amid the variables. In this connection, regression investigation can be applied.

**Table 5:** Output of Johansen cointegration test.

Variables	Pedroni panel cointegration test			Kao panel cointegration test
	MPP t	PP t	ADF t	ADF
economic system	−5.217 (0.02)	5.103 (0.00)	−4.496 (0.00)	−1.310 (0.00)
economic effectiveness	−8.527 (0.00)	−6.307 (0.03)	−5.931 (0.01)	0.720 (0.02)
Environmental Quality	−7.604 (0.01)	−7.083 (0.00)	−7.201 (0.00)	1.416 (0.02)

The Harris–Tzavalis test is executed to investigate whetherfixedproducts exist or not between variables. The Harris–Tzavalis test outcomes are presented in Table (6). Based on the output of the Harris–Tzavalistest from Table (6), for this result, the researcher should apply a fixed or arbitrary effect model to measure the coefficient of the method when the environmental quality protection and economic systems are investigated as dependent variables. When economic significance is analysed as the response variable, the selected or arbitrary random effect method should be used.

**Table 6:** Harris–Tzavalis-test.

Sustainable economic growth	Chi-Square (Χ^2^) Tests	Probability
economic system	17.26	0.002
economic effectiveness	6.94	0.076
Environmental Quality	19.04	0.004

### 4.4 Regression analysis output

From Table seven, the output of the model (1), (4) and (7) shows that green finance has a substantial positive influence on environmental quality protection management (α = 0.0.009**, p < 0.01), economic effectiveness (α = 0.034***, p < 0.05) and economic system (α = 0.014***, p < 0.05), correspondingly, considering to hypothesis one, two and three. Consequently, the outcomes authorize that green finance can progress sustainable economic growth from selected three magnitudes variables, which also reproduces the efficiency of correlated strategy in enlightening the sustainable economic development (see Table 9). Furthermore, model number 3 explains the controlling role of financial technology in influencing the association between green finance and environmental quality protection management.

**Table-7:** Output of regression analysis – Sustainable economic growth.

Variables	Environmental Quality			Economic Effectiveness			Economic System		
	M-1	M-2	M-3	M-4	M-5	M-6	M-7	M-8	M-9
Green finance	0.009** (0.002)	0.0011*** (0.002)	0.010*** (0.003)	0.034*** (0.004)	0.036*** (0.007)	0.047** (0.007)	0.014*** (0.002)	0.017*** (0.004)	0.012***(0.004)
Fintech		−0.001 (0.001)	−0.002 (0.004)		0.013* (0.002)	0.013*** (0.002)		0.004*** (0.002)	0.002*** (0.001)
Green finance*fintech			0.008*** (0.003			0.002 (0.004)			0.005** (0.008)
Energy consumption	0.001 (0.004)	0.002 (0.004)	0.004 (0.005)	0.047* (0.009)	0.05*** (0.009)	0.053*** (0.009)	0.079*** (0.002)	0.081** (0.004)	0.099** (0.007)
GDP per Capita	0.053*** (0.003)	0.052** (0.001)	0.057*** (0.004)	0.059*** (0.011)	0.061*** (0.008)	0.049** (0.008)	0.004 (0.003)	0.003 (0.002)	0.005 (0.005)
Fiscal expenditure	−0.001 (0.024)	−0.014 (0.027)	−0.012 (0.025)	−0.134*** (0.057)	−0.183 (0.056)	−0.159*** (0.091)	−0.083** (0.44)	−0.052** (0.39)	−0.086** (0.044)
Education %	0.237 (0.225)	0.213 (0.232)	0.208 (0.189)	−0.927** (0372)	−1.259*** (0.357)	−1.083*** (0.437)	−0.002 (0.137)	−0.026 (0.243)	−0.043 (0.248)
Constant Value	−0.247** (0.260)	−0.261*** (0.027)	−0.210*** (0.028)	−0.852** (0.081)	−0.573*** (0.082)	0.049** (0.103)	−0.048*** (0.046)	−0.438*** (0.045)	−0.648*** (0.049)
Observation	328	328	328	328	328	328	328	328	328
R^2^	0.231	0.311	0.362	0.217	0.247	0.261	0.317	0.362	0.471
F-statistics	89.01***	76.00**	56.41**	124.50**	153.26***	164.41**	217.36**	201.73***	185.78**

**Table 8:** Two-step generalized method of moments (GMM) estimators of Sustainable economic growth.

Variables	Environmental Quality	Economic Effectiveness	Economic System
	M-1	M-2	M-3
Green finance	0.019*** (0.005)	0.103** (0.017)	0.027** (0.002)
Fintech	−0.001 (0.003)	0.011*** (0.007)	0.009*** (0.001)
Green finance*fintech	0.007** (0.002)	0.008 (0.006)	0.037** (0.031)
Energy consumption	0.006*** (0.002)	0.054*** (0.013)	0.084*** (0.007)
GDP per capita	0.068*** (0.002)	0.086*** (0.013)	0.012 (0.006)
Education %	0.349 (0.241)	−0.948*** (0.528)	0.104 (0.281)
Fiscal expenditure	−0.024** (0.034)	−0.196** (0.104)	−0.021*** (0.029)
Constant Value	−0.493*** (0.327)	1.258 (0.837)	−0.734*** (0.106)
wald χ2 tests	306.54***	315.16***	749.15***
R^2^	0.237	0.375	0.518

**Table 9:** To validate the study hypotheses.

S·NO		Hypotheses	P Value	Accept/Reject
1	H1A	Green finance is positively correlated to environmental quality protection.	β = 0.014, p < 0.05	Accept
2	H1B	Green finance is positively associated with high efficiency of economic development.	(β = 0.070, p < 0.01	Accept
3	H1C	Green finance is positively correlated to the development of the economic system.	(β = 0.021, p < 0.01	Accept
4	H2A	Fintech significantly balanced the impact of green finance on environmental quality protection.	(β = 0.010, p < 0.01	Accept
5	H2B	Fintech significantly balanced the impact of green finance on economic effectiveness.	(β = 0.004, SE = 0.008)	Reject
6	H2C	Fintech significantly balanced the impact of green finance on the economic system.	(β = 0.012, p < 0.05	Accept

In the view of table value (Table 7), the interface tenure is suggestively positive (α = 0.008***, p < 0.05), signifying that fintech progresses the constructive impact of green finance on environmental quality protection management. Based on model 3, hypothesis 4 shows that it the significant and positive. In this connection, green finance and environmental quality protection variables support hypothesis 4. The other side is that financial technology enables the result of green finance by efficiently fascinating green capital from the non-public sector and retail investors. However, the development of fintech indicates that the adverse and irrelevant or insignificant.

Concerning the controlling variable of fintech on the connection between green finance and economic effectiveness, model number (6) indicates that divergent to the research potentials, financial technology does not suggestively support the association between green finance and economic significance (α = 0.002, SE = 0.004), the green finance and economic effectiveness values are not significant and unsporting to the hypothesis 5 (see Table 9). One of the major issues for India is that green finance and fintech are created limited space for crowdfunding and energy trading. In this connection, the growth of fintech in the green finance development area is still in its infancy, and its suggestions are a rational justification for the unpredicted insignificant controlling effect studied in previous literature. Additionally, in relationships of the economic system, as projected, model number (8) illustrates that fintech certainly has a better and more significant association between green finance and the financial system (α = 0.005**, p < 0.01). Hence, hypothesis 6 indicates a significant and positive impact on sustainable economic growth, (see Table 9).

In the view of the control variable, the output from Table 7, the use of energy from the last three years hasindicated the substantial and positive impact with related models of the economic system and effectiveness are response variables and do not have any significant or positive impact on the environmental quality protection management. Due to this cause, more energy usage in the preceding years may contribute to sustainable economic development but have an unfavourable effect on environmental quality protection. The output from Table 7 shows the undesirable and adverse impact on sustainable economic growth's environmental, and ecological quality management. GDPPC (gross domestic product per capita) shows substantial positive outcomes on environmental quality protection management and economic effectiveness but not on the financial system. Educational per cent has a negative association with economic significance. This specifies that, with the economy's development, it needs a better utilization of resources to augment productivity. The measure of fiscal expenditure is also negatively associated with economic effectiveness and system, which also suggests the further operative use of capital with the growth of a sustainable economy.

### 4.5 Robustness check: endogeneity problem

The generalized method of moments (GMM) is a statistical method that combines observed economic data with information on population moment conditions to produce estimates of the unknown parameters of this economic model. From Table 8, the research paper examines the potential endogeneity of the descriptive variables. In the meantime, the endogeneity of the variables defines whether a variable is associated with unobservable regression error terms analysis. In this regard, no direct statistical method can fully tenacity the problem.

Nevertheless, if the researcher does not address the endogeneity issues, the regression outcome could be e biased and mislead examination conclusions. In this connection, the present research paper has considered indirect tests to examine the endogeneity apprehension. Based on Table 8, the report explores the unified series of Indian state-level control variables (energy consumption, GDPPC, % of educational level, fiscal expenditure) while assessing the research method. The comprised control variables are equally academically and analytically pertinent, which decreases the casual of endogeneity outcomes from misplaced variables. Another, the paper applies a generalized two-step method of moments (GMM) estimators of Sustainable economic growth employing device variables. The form applied lagged green finance and financial technology of each Indian state as device variables for each state's current green finance and financial technology, correspondingly. Using the lagged variable satisfies the criteria during the sample period. (1) It must be highly associated with the endogenous variable during the sample period of the study, and (2) it is not associated with the error terms. As the output from Table 8, the outcomes show no significant variances from the original regression outcomes, signifying the robustness of the present study results.

## 5 Limitations of the study

This research paper also has some limitations, primarily regarding inadequate data accessibility, making it hard to analyse the heterogenous issues that ultimately impact the research model. Green finance comprehends a variety of themes, including green bonds, green insurance, and green credit. It is interesting to get the above statistics because of inadequate information sharing. Our examination cannot determine the significance of numerous green financing strategies on ecological sustainability. Academic research is progressive, making it informal to gather pertinent data and comportment future studies on how various green financing methods affect a sustainable economy. Sustainable development is broad and includes social, environmental, and economic spheres. We focus on environmental sustainability, but future studies might also consider how green financing affects corporate environmental behaviour in other areas to enhance the findings. CO2 emissions are only a small part of developing nations' complex environmental contamination problem.

## 6 Conclusion and policy implications

The current research paper used the Indian states' panel data from 2010 to 2021. It developed a conceptual framework to analyse the instrument of financial technology and green finance in accomplishing the objectives of sustainable economic growth. The paper chose the five parameters to assess green finance's magnitude and composite them using a general principal component analysis model (GPCAM). Simultaneously, the researcher developed sustainable economic growth parameters from 3 proportions, (1) environmental quality protection (2) economic effectiveness, and (3) economic system. The study's original findings presented that green finance lengthily contributes to sustainable economic growth by an undoubtedly significant impact on all three proportions or aspects (1) environmental quality protection (2) economic effectiveness, and (3) economic system), which analytically confirm the constructive outcome of green finance. In addition, financial technology enables green finance positive and considerable influence on environmental quality protection and economic system characteristics. However, fintech does not control the association between green finance and its economic effect. This is the reason behind the sufficient areas of green finance that fintech comprises in India.

The present research paper has drawn policy implications based on the findings: (1) financial managers need to accelerate the growth of financial technology with green financeand supervisors should boost fintech firms to energetically engage in several areas of green finance projects and environmental quality safeguarding enterprises that enable sustainable economic growth. (2) The green finance program and assessment arrangement are still in the opening stage, where comprehensive execution standards remain inadequate. Policymakers need to plan an ecological revelation background, while the government of India should improve its direction of state governments to expand the effectiveness of green finance. (3) operative policy involvement in the short-term and long-term plan desires to be framed to endorse the progress of green finance. Becauseof India's fiscal restraints, over 45% of the green finance will be received from the private segment to accomplish green growth.

## Author contribution statement

Dr Nenavath Sreenu Conceived and designed the experiments; Performed the experiments; Analysed and interpreted the data; Contributed reagents, materials, analysis tools or data; Wrote the paper.

## Funding

This research did not receive any specific grant from funding agencies in the public, commercial, or not-for-profit sectors.

## Ethical approval and consent to participate

No human participants were involved in this research study

## Consent to publish

Manuscript does not contain data from any person and is not applicable,

## Data availability statement

Data will be made available on request

## Pre-print service

The manuscript not submitted to a preprint server prior to submission

## Declaration of competing interest

The authors declare that they have no known competing financial interests or personal relationships that could have appeared to influence the work reported in this paper.
